# A 16-Year Longitudinal Study of Long-term Clinical and Radiographic Outcomes in Dorsal Proximal Interphalangeal Joint Fracture-Dislocations

**DOI:** 10.1177/15589447261424449

**Published:** 2026-03-16

**Authors:** Panu H. Nordback, Marjut Westman, Eero Waris

**Affiliations:** 1 Helsinki University Central Hospital and University of Helsinki, Finland; 2 Päijät-Häme Central Hospital, Lahti, Finland

**Keywords:** finger injury, fracture-dislocation, long-term outcome, post-traumatic osteoarthrosis, proximal interphalangeal joint

## Abstract

**Background:**

Dorsal fracture-dislocations of the proximal interphalangeal (PIP) joint often lead to stiffness, pain, and post-traumatic osteoarthritis (PTOA). This study evaluated long-term clinical outcomes and radiographic PTOA following surgical treatment of unstable PIP fracture-dislocations.

**Methods:**

We conducted a retrospective cohort study of 25 patients (27 fingers) treated with extension block pinning for unstable dorsal PIP fracture-dislocations between 2000 and 2009. Follow-ups in 2010 and 2021 assessed active range of motion (AROM) in PIP and distal interphalangeal (DIP) joints, grip strength, and pain. Postoperative radiographs were analyzed for articular surface involvement and step-off. Post-traumatic osteoarthritis was graded using Kellgren-Lawrence, Kallman, and Osteoarthritis Research Society International scales by 3 blinded hand surgeons, with intraobserver and interobserver reliability assessed.

**Results:**

Over a follow-up of up to 16 years, PIP AROM remained stable. Patients aged ≥45 had reduced PIP AROM and more frequent pain. Greater pain was also seen in those with >0.5 mm articular step-off or advanced radiographic osteoarthritis (OA). Osteoarthritis progression was associated with reduced PIP AROM and residual step-off. The Kallman scale showed the most significant OA progression, especially with ≥50% joint surface involvement. Osteoarthritis grading showed substantial intraobserver and interobserver reliability.

**Conclusion:**

Long-term function after extension block pinning of dorsal PIP joint fracture-dislocations remained, though age over 45 years and residual step-off predicted reduced AROM, pain, and OA progression.

## Introduction

Post-traumatic osteoarthritis (PTOA) is a progressive condition triggered by acute joint injury, which sets off a cascade of events that can lead to ongoing damage to the articular surface over time. The pathogenetic mechanisms of PTOA remain partially understood, where altered biomechanics and molecular processes are considered key contributors.^1,2^ Different joints and even specific regions within the same joint exhibit varying tolerance to articular changes.^3^

In weight-bearing joints like the knee, PTOA commonly develops following intra-articular fractures, meniscal tears, ligament injuries, and chondral damage.^4-7^ In the wrist, conditions such as carpal ligament injuries, nonunion of the carpal bones, or distal radius malunion can lead to misalignment, altered kinematics, and abnormal load distribution, increasing the risk of radiographic PTOA. Similarly, in the trapeziometacarpal and interphalangeal joints of the fingers, intra-articular fractures, such as first metacarpal base fractures and bony mallet fingers, can contribute to the development of radiographic PTOA.^8,9^ However, the relationship between radiographic and clinical PTOA remains incompletely established.^8-12^

Proximal interphalangeal (PIP) joint fracture-dislocations are among the most disabling finger injuries, often stated to result in prolonged pain, stiffness, and long-term functional impairment.^13^ Proximal interphalangeal joint fracture-dislocations affecting the middle phalanx base can be classified into either dorsal or volar dislocations based on the direction of middle phalanx displacement, where loss of the volar plate and collateral ligaments (dorsal) or central slip (volar) support lead to joint subluxation.^14^ The annual incidence of dorsal PIP fracture-dislocations has been estimated to be 9/100 000 persons.^15^ Surgical intervention is often considered to be indicated for fractures with greater than 30% to 50% articular involvement in the base of the middle phalanx, where joint instability arises from the loss of both articular and ligamentous support.^14,16^ While articular incongruence may increase the risk of radiographic PTOA, it does not always correlate with clinical outcomes.^17^ To date, only a few large, long-term studies have explored the development of clinical and radiographic PTOA after hand and finger fractures, but not regarding dorsal PIP fracture-dislocations.^8,9,17^

Radiographically, osteoarthritis (OA) is characterized by joint space narrowing, subchondral sclerosis, osteophyte formation, and joint subluxation.^18^ Several standardized scoring systems, including the Kellgren-Lawrence scale, Kallman scale, and the Osteoarthritis Research Society International (OARSI) atlas, are available to assess radiographic OA.^19,20^ However, comparative data on their applicability and their relationship to clinical manifestations of PTOA in the hand is limited.^21^ The clinical presentation and progression of PTOA are variable, but it typically manifests as pain, swelling, stiffness, and functional disability, which tend to worsen over time.^21^

This study aimed to assess the progression of both clinical and radiographic PTOA following surgically treated unstable dorsal PIP joint fracture-dislocations, with mid- to long-term follow-up spanning 16 years. Specifically, we sought to determine whether PTOA progressed in the non-weight-bearing PIP joints over time and to identify potential associations between demographic factors, articular incongruency, radiographic OA scores, and clinical outcomes. In addition, we evaluated the reliability of the 3 most commonly used radiographic OA scoring methods and OA score correlation with seen clinical results to assess if any of them would be superior to the others in evaluating finger arthrosis.

## Materials and Methods

### Patients

The study was approved by the hospital’s institutional review board (HUS/1980/2020, HUS/234/2020), and patient consent was obtained in accordance with the Declaration of Helsinki. We identified a retrospective cohort of 53 patients with 55 dorsal fracture-dislocations of the PIP joint, all treated with extension block pinning and postoperative rehabilitation between 2000 and 2009, as previously reported (Supplementary Figure 1).^22^ Patients were invited to participate in a follow-up study, which included a questionnaire, clinical examination, and plain radiographs, conducted in 2010 and 2021. The mean interval between the operation and the first follow-up was 5 years (SD = 3, range = 1-11 years), and the second follow-up occurred after a mean of 16 years (SD = 3, range = 12-21 years), resulting in a mean of 11 years (range = 10-12 years) between the 2 follow-up evaluations. Of the original cohort, 25 patients (27 injured fingers) attended both follow-up visits, including radiographic assessments. These 25 patients were included in the study. Of the original cohort, 1 patient moved abroad, 5 deceased, 8 refused to participate, 8 were unattainable, and 6 had incomplete data regarding clinical outcomes and/or follow-up radiographs. Detailed patient demographics and injury data are provided in Table 1. Three patients had associated distal phalanx injuries: 2 bony mallet fractures and 1 distal phalanx shaft fracture, the latter of which required subsequent distal interphalangeal (DIP) joint fusion. These patients with associated injuries were excluded from the DIP joint measurements.

**Table 1.** Patient Demographics.

Demographic	Value
Patients (n)	25
Injured fingers (n)	27
Patient age at follow-up (years; median, range)	57 (33-84)
Sex, male/female (n)	17/8
Injured hand dominance, dominant/non-dominant (n)	12/13
Time interval from injury to operation (days; median, range)	8 (1-27)
Injured finger (n)	
Index	4
Long	8
Ring	12
Little	3
Fracture involvement of the articular surface of the middle phalanx (%: median, range)	48 (40-65)
Associated injuries (n)	
Bony mallet finger	2
Distal phalanx shaft fracture	1
Removal of the extension block pin (days; median, range)	23 (14-33)

### Follow-up Evaluation

Outcome measures, including clinical examinations and radiographs, were recorded during follow-ups in 2010 and 2021.^17,22^ Objective measurements were performed by a single physiotherapist. The active range of motion (AROM) of the DIP and PIP joints was measured using a goniometer with the nearest 5-degree accuracy. The tip-to-palm distance (in mm) at full flexion of the injured finger was measured from the distal palmar crease. Grip strength and injured fingertip pinch strength (injured finger − thumb) were assessed using sustained measurement with Jamar hand dynamometer (Saehan Corporation, Seoul, South Korea) and a pinch gauge (Baseline pinch gauge, Alimed Corporation, Dedham, Massachusetts), respectively. For pain assessment, patients were asked to rate the overall pain in the injured finger using the Visual Analog Scale (VAS) during the first follow-up and the 11-point Numeric Rating Scale (NRS) (0 = no pain, 10 = worst possible pain) during the second follow-up. In 2 cases, objective measures at the second follow-up could not be assessed due to remote evaluations, but pain questionnaires and radiographs were available.

### Radiographic Evaluation

Preoperative radiographs were used to calculate the extent of fracture involvement on the articular surface of the base of the middle phalanx, expressed as a percentage of the total joint surface.^22^ On immediate postoperative radiographs, the residual articular step-off was measured by evaluating the maximum step-off on the articular surface. Follow-up radiographs were assessed for the presence of OA using the Kellgren-Lawrence, Kallman, and OARSI scoring atlas.^23^ Briefly, in the Kellgren-Lawrence scale, presence and size of osteophytes and joint space narrowing are evaluated, whereas in the Kallman and OARSI methods osteophytes, joint space narrowing, subchondral sclerosis and cysts, lateral deformity, and collapse of central joint cortical bone (Kallman) or joint erosion (OARSI) are evaluated. All radiographs were evaluated and scored in a blinded manner by 3 hand surgeons (PHN, MW, EW) in 2 rounds, using both posterior-anterior and lateral views.

### Statistical Analysis

Numerical data are presented as median values with 25th to 75th percentiles, unless stated otherwise (patient demographics in Table 1). Group comparisons were performed using Student *t* test or the Mann-Whitney *U* test for continuous variables, and Fisher exact test for categorical variables. Comparisons between the injured and contralateral sides were made using the paired samples *t* test. Results of the *t* tests are presented as the mean difference with 95% confidence intervals. A 2-tailed *P*-value of <.05 was considered statistically significant. If the difference between groups was different at different times, the *P*-value is given as group-by-time interaction effect. Associations between continuous variables were assessed using Spearman correlation coefficient (ρ). Correlation coefficients were classified as follows: strong (0.75-1), moderate (0.5-0.75), weak (0.25-0.5), or negligible (0-0.25). For intraobserver and interobserver reliability, intraclass correlation coefficients (ICCs) were calculated and categorized based on the strength of agreement: poor (<0.00), slight (0.00-0.20), fair (0.21-0.40), moderate (0.41-0.60), substantial (0.61-0.80), or almost perfect (0.81-1.00).^24^

## Results

### Clinical Outcomes

Table 2 presents the clinical outcomes, including the AROM of the PIP and DIP joints at 2 follow-up time points. Grip strength difference also showed a significant reduction compared with the uninjured contralateral hand (*P* = .03) between the 2 time points. However, there were no statistically significant differences (*P* > .05) in PIP joint AROM, tip-to-palm distance, tip pinch strength, or pain between the 2 follow-up evaluations.

**Table 2.** Clinical Outcome Measures at the two Follow-up Time Points (Median; 25th-75th Percentile).

Parameter	First follow-up	Second follow-up	Difference (95% CI)	*P*-value
PIP ROM (°)	85 (65 to 95)	80 (55 to 95)	0.51 (−5.4 to 6.5)	.86
DIP ROM (°)	80 (60 to 85)	70 (50 to 76)	5.5 (−7.3 to 18)	.41
PIP + DIP ROM (°)	165 (136 to 171)	155 (116 to 173)	−10.2 (−36 to 16)	.44
Tip-to-palm (mm)	4 (0 to 15)	0 (0 to 25)	−0.85 (−4.4 to 2.7)	.31
Grip strength difference (kg)	−4.5 (−9.0 to 0)	−1.0 (−4.0 to 2.0)	−4.0 (−7.7 to −0.35)	**.03**
Tip pinch strength difference (kg)	0 (−2.0 to 0.5)	0 (−0.6 to 0.8)	−0.4 (−1.3 to 0.45)	.33
Pain	0 (0 to 2.9)^a^	0 (0 to 1.0)^b^	0.25 (−0.25 to 0.70)	.31

*Note.* Difference = difference between means; CI = confidence interval; PIP = proximal interphalangeal; ROM = range of motion; DIP = distal interphalangeal; NRS = Numeric Rating Scale. *p* < 0.05 in bold.

a Visual Analog Scale (VAS).

b Numeric Rating Scale (NRS).

The AROM of the PIP joint was significantly better (*P* < .05) at both follow-up time points in patients who were younger than 45 years at the time of injury. A trend (*P* < .1) regarding PIP joint AROM and age was also observed for the group-by-time interaction between follow-up time points (Table 3). Neither articular step-off, joint surface involvement, injured side dominance, nor patients’ sex were associated with PIP joint AROM in our cohort (Table 3).

**Table 3.** Effect of Various Parameters on PIP Joint Range of Motion (Median; 25th-75th Percentile).

Parameter	Subgroup	Number of fingers (n^1st^/n^2nd^)	First follow-up	Second follow-up	*P*-value (time × group)	Difference (95% CI) *P*-value (first follow-up)	Difference (95% CI) *P*-value (second follow-up)
Step-off	≤0.5 mm >0.5 mm	15/14 12/11	85 (75-95) 72 (55-95)	90 (60-105) 75 (55-95)	>.90	14 (−1.4 to 29) .074	14 (−1.0 to 29) .066
Joint surface involvement	<50% ≥50%	16/15 11/10	88 (71-95) 77 (55-95)	80 (55-105) 80 (55-95)	.54	9 (−11 to 29) .37	5 (−15 to 25) .61
Handedness	Non-dominant Dominant	14/14 13/11	85 (55-95) 85 (75-95)	83 (45-105) 75 (55-95)	.80	6 (−13 to 25) .52	5 (−15 to 24) .63
Age (at the time of injury)	<45 ≥45	15/13 12/12	95 (80-97) 66 (55-83)	95 (80-105) 58 (43-80)	.084	20 (3.4 to 37) **.020**	30 (13 to 47) **.001**
Gender	Male Female	19/18 8/7	85 (75-95) 74 (58-98)	88 (60-105) 65 (55-95)	>.90	−4.3 (−25 to 17) .68	−3.9 (−25 to 18) .71

*Note.* Time × group = group by time interaction; CI = confidence interval; difference = difference between means. *p* < 0.05 in bold.

The VAS pain score was significantly higher (*P* = .048) at the first follow-up in patients with a residual articular step-off greater than 0.5 mm postoperatively (Table 4). Patients younger than 45 years at the time of injury had a significantly lower VAS pain score (*P* = .024) at the first follow-up compared with patients aged 45 or older. No significant differences in pain were found in relation to joint surface involvement, hand dominance, or patients’ sex (*P* > .05) between the 2 follow-up time points. A moderate positive correlation was found between pain (VAS) and all OA scoring methods at the first follow-up: Kellgren-Lawrence (ρ = 0.54, *P* = .01), Kallman (ρ = 0.57, *P* = .003), and OARSI (ρ = 0.56, *P* = .003).

**Table 4.** Effect of Various Parameters on Pain (Median; 25th-75th Percentile).

Parameter	Subgroup	First follow-up^a^	Second follow-up^b^	*P*-value (time × group^a,b^)	Difference (95% CI) *P*-value (first follow-up^a^)	Difference (95% CI) *P*-value (second follow-up^b^)
Step-off	≤0.5 mm >0.5 mm	0 (0-0) 2 (0-3)	0 (0-1) 0.5 (0-1.5)	.087	−1.1 (−2.3 to −0.0097) **.048**	−0.30 (−1.4 to 0.78) .58
Joint surface involvement	<50% ≥50%	0 (0-3) 0 (0-1)	1 (0-1) 0 (0-1)	.85	0.59 (−0.67 to 1.9) .35	0.69 (−0.55 to 1.9) .27
Handedness	Non-dominant Dominant	0 (0-2.9) 0 (0-2)	0 (0-1) 1 (0-1)	.39	−0.12 (−1.4 to 1.1) .85	0.30 (−0.92 to 1.5) .62
Age (at the time of injury)	<45 ≥45	0 (0-0) 2 (0-3)	1 (0-1) 0 (0-1)	**.024**	−1.30 (−2.6 to −0.19) **.024**	−0.30 (−1.5 to 0.87) .61
Gender	Male Female	0 (0-2.9) 0 (0-2)	1 (0-1) 0 (0-1)	.71	−0.27 (−1.6 to 1.1) .69	−0.07 (−1.4 to 1.3) >.9

*Note.* Time × group = group by time interaction; CI = confidence interval; difference = difference between means. *p* < 0.05 in bold.

a Visual Analog Scale (VAS).

b Numeric Rating Scale (NRS).

### Radiographic Evaluation

On primary radiographs, the median articular surface involvement was 48% (range = 40%-65%), and the median postoperative residual articular step-off was 0.5 mm (range = 0.0-1.4 mm). At the first follow-up, median OA score for the PIP joint was 2 (range = 1-2), 2 (1-3), and 2 (1-3) for Kellgren-Lawrence, Kallman, and OARSI, respectively. At the second follow-up, scores were 2.5 (range = 1.5-4), 2 (1-2), and 2.5 (1.5-3.5). Significant differences were observed with the Kallman method (*P* = .019). Moderate negative correlations were found between radiographic OA and PIP joint AROM at both follow-ups. At the first follow-up, correlations were Kallman ρ = −0.44 (*P* = .02), OARSI ρ = −0.50 (*P* = .01), and Kellgren-Lawrence ρ = −0.50 (*P* = .02). At the second follow-up, correlations were Kallman ρ = −0.64 (*P* < .001), OARSI ρ = −0.67 (*P* < .001), and Kellgren-Lawrence ρ = −0.71 (*P* < .001).

All OA scores were significantly higher (*P* < .05) when the postoperative residual step-off exceeded 0.5 mm (Tables 5-7). No association was found between hand dominance and OA scores. In patients aged 45 years or older at the time of injury, the OA score was significantly higher (*P* = .045) at the first follow-up according to the Kellgren-Lawrence method, and a similar trend was observed at the second follow-up (*P* = .028) (Table 6). No differences in OA scores were found based on patients’ sex.

**Table 5.** Impact of Fracture and Patient Variables on OA Using the Kallman Method (Median; 25th-75th Percentile).

Parameter	Subgroup	First follow-up	Second follow-up	*P*-value (time × group)	Difference (95% CI) *P*-value (first follow-up)	Difference (95% CI) *P*-value (second follow-up)
Step-off	≤0.5 mm >0.5 mm	1.0 (1.0-2.0) 3.0 (2.5-3.5)	1.5 (1.5-3.0) 3.0 (2.5-5.0)	.38	−1.4 (−2.3 to −0.4) **.005**	−1.1 (−2.0 to −0.1) **.024**
Joint surface involvement	<50% ≥50%	3.0 (1.0-3.0) 2.0 (1.0-3.0)	3.0 (1.8-4.3) 2.5 (1.5-3.5)	.83	0.2 (−1.3 to 1.7) .76	0.3 (−1.2 to 1.8) .68
Handedness	Non-dominant Dominant	2.0 (1.0-3.0) 2.0 (1.0-3.0)	2.3 (1.5-4.0) 3.0 (1.5-3.0)	.90	−0.2 (−1.6 to 1.3) .79	−0.2 (−1.7 to 1.2) .74
Age (at the time of injury)	<45 ≥45	1.0 (1.0-3.0) 3.0 (2.0-3.0)	2.0 (1.5-3.0) 3.0 (2.5-4.3)	.62	−1.1 (−2.5 to 0.2) .10	−1.3 (−2.7 to 0.07) .062
Gender	Male Female	2.0 (1.0-3.0) 2.5 (2.0-3.5)	2.0 (1.5-4.0) 3.0 (2.3-4.3)	>.90	0.2 (−1.3 to 1.8) .78	0.3 (−1.3 to 1.8) .74

*Note.* OA= osteoarthritis; time × group = group by time interaction; CI = confidence interval; difference = difference between means. *p* < 0.05 in bold.

**Table 6.** Impact of Fracture and Patient Variables on OA Using the Kellgren-Lawrence Method (Median; 25th-75th Percentile).

Parameter	Subgroup	First follow-up	Second follow-up	*P*-value (time × group)	Difference (95% CI) *P*-value (first follow-up)	Difference (95% CI) *P*-value (second follow-up)
Step-off	≤0.5 mm >0.5 mm	1.0 (1.0-2.0) 2.0 (2.0-2.8)	1.0 (1.0-2.0) 2.0 (2.0-2.8)	.43	−1.0 (−1.4 to −0.6) **<.001**	−0.9 (−1.3 to −0.5) **<.001**
Joint surface involvement	<50% ≥50%	2.0 (1.0-2.0) 2.0 (1.0-2.0)	2.0 (1.0-2.0) 2.0 (1.0-2.0)	.51	−0.05 (−0.8 to 0.7) .89	0.07 (−0.6 to 0.8) .84
Handedness	Non-dominant Dominant	2.0 (1.0-2.0) 2.0 (1.0-2.0)	2.0 (1.0-2.0) 2.0 (1.0-2.0)	.54	0.1 (−0.6 to 0.8) .74	0.002 (−0.7 to 0.7) >.90
Age (at the time of injury)	<45 ≥45	1.0 (1.0-2.0) 2.0 (2.0-2.0)	1.0 (1.0-2.0) 2.0 (2.0-2.3)	.32	−0.5 (−1.2 to 0.1) .09	−0.7 (−1.4 to −0.09) **.03**
Gender	Male Female	2.0 (1.0-2.0) 2.0 (1.5-2.5)	2.0 (1.0-2.0) 2.0 (2.0-2.5)	.30	0.2 (−0.5 to 0.9) .59	0.4 (−0.3 to 1.1) .28

*Note.* OA = osteoarthritis; time × group = group by time interaction; CI = confidence interval; difference = difference between means. *p* < 0.05 in bold.

**Table 7.** Impact of Fracture and Patient Variables on OA Using the Osteoarthritis Research Society International Method (Median; 25th-75th Percentile).

Parameter	Subgroup	First follow-up	Second follow-up	*P*-value (time × group)	Difference (95% CI) *P*-value (first follow-up)	Difference (95% CI) *P*-value (second follow-up)
Step-off	≤0.5 mm >0.5 mm	1.0 (0.5-2.0) 3.0 (2.8-4.0)	1.5 (1.0-3.0) 3.0 (2.3-4.8)	.1	−1.6 (−2.6 to −0.6) **.007**	−1.0 (−2.0 to −0.03) **.04**
Joint surface involvement	<50% ≥50%	2.3 (1.0-3.5) 2.0 (1.0-3.0)	3.0 (1.5-4.3) 2.0 (1.5-3.5)	.67	0.08 (−1.5 to 1.6) .92	0.2 (−1.3 to 1.8) .75
Handedness	Non-dominant Dominant	2.0 (1.0-3.0) 2.5 (1.5-3.0)	2.3 (1.5-4.0) 3.0 (1.5-3.0)	.18	0.2 (−1.4 to 1.7) .84	−0.3 (−1.8 to 1.2) .65
Age (at the time of injury)	<45 ≥45	1.5 (0.5-3.0) 2.8 (2.0-4.0)	1.5 (1.0-3.0) 3.0 (2.5-4.3)	.54	−1.2 (−2.6 to 0.2) .06	−1.4 (−2.8 to 0.009) **.05**
Gender	Male Female	2.0 (1.0-3.0) 3.0 (2.3-4.0)	2.0 (1.5-3.5) 3.0 (2.5-4.3)	.53	0.7 (−1.0 to 2.3) .40	0.4 (−1.2 to 2.0) .61

*Note.* OA = osteoarthritis; time × group = group by time interaction; CI = confidence interval; difference = difference between means. *p* < 0.05 in bold.

The interobserver reliability for all 3 OA scoring methods was substantial (Kellgren-Lawrence ICC = 0.619; Kallman ICC = 0.718; OARSI ICC = 0.769) for detecting PIP joint OA. The OARSI method demonstrated the highest intraobserver reliability, approaching “almost perfect” agreement among all observers (ICC = 0.831-0.857). In comparison, the Kellgren-Lawrence (ICC = 0.646-0.788) and Kallman (ICC = 0.728-0.828) methods also showed at least substantial intraobserver reliability.

## Discussion

In this retrospective longitudinal study of patients who underwent extension block pinning for unstable dorsal PIP fracture-dislocations, we observed radiographic progression of OA in the affected joint between mid- to long-term follow-up. Interestingly, while radiographic evidence of OA progressed using the Kallman scale, clinical symptoms—such as AROM and pain levels in the PIP joint—did not worsen. However, radiographic OA was associated with a reduction in AROM at both mid- and long-term follow-ups, and increased pain levels were noted only at the mid-term follow-up. These findings align with previous studies on intra-articular distal radius fractures and mallet finger injuries, where radiographic PTOA progressively worsens over time and correlates with decreased range of motion.^9,25,26^ In addition, our study found a significant decrease in the grip strength difference between the injured and healthy sides between the mid- and long-term follow-up assessments. Previous research has demonstrated that recovery of hand grip strength after distal radius fractures can extend over several years, but our observation could also be aging related, with grip strength being decreased over time and possibly interfered by symptomatic OA.^27^

In this study, older age at the time of injury and residual articular step-off were identified as prognostic factors that influence negative clinical outcomes. Specifically, reduced PIP joint AROM was associated with patients aged 45 years and older. In addition, elevated pain levels were observed at the first follow-up in patients aged ≥45 years and those with postoperative articular step-off greater than 0.5 mm. However, this pain association was no longer evident at the second follow-up, 11 years later, suggesting a natural course of joint regeneration and a subsequent reduction in pain over time. To the best of our knowledge, this is the first study to identify prognostic factors that influence long-term clinical outcomes following finger fractures. A step-off greater than 2 mm in distal radius fractures has been linked to radiographic PTOA, but its impact on clinical outcomes remains unclear.^25^ It can be hypothesized that the smaller joints of the hand, such as the PIP joint, might have less tolerance for articular step-off.

Our study explored the possible factors contributing to the progression of radiographic OA. We observed a significant increase in the Kallman OA score between follow-up time points in patients with residual articular step-off (>0.5 mm) or those with more than 50% articular surface involvement in the original fracture. Both increased articular surface involvement and step-off contribute to joint incongruence, resulting in abnormal loading of the cartilage and subchondral bone. This excessive mechanical load can exceed the bearing capacity of hyaline cartilage, leading to progressive cartilage degeneration—a phenomenon well documented in weight-bearing joints such as the hip and knee.^28^ It is important to note that different joints, and even distinct regions within the same joint, may have varying tolerance levels to post-traumatic articular step-off.^3^ In our study, patients aged more than 45 at the time of injury were found to have a significantly higher risk for radiographic PTOA, suggesting that biological factors may play a role in the progression of PTOA. Thus, some degree of OA progression could potentially be influenced by aging, which was our original reason to set the age cutoff to 45 years. In the context of weight-bearing joint injuries, previous research has identified several prognostic factors for clinical and radiographic PTOA, including obesity, age (>40 years), malalignment, instability, and residual articular incongruence.^29-31^

Several radiographic classification systems have been developed to assess the severity of OA. In our study, we selected the most widely used systems for evaluating hand OA.^20^ The Kellgren and Lawrence scale has long been a cornerstone in the literature for assessing OA on plain radiographs.^19^ However, Kallman modified this classification by separately evaluating osteophytes and joint space narrowing, making it more sensitive than the original Kellgren-Lawrence scale.^32^ In our study, the Kallman method demonstrated greater sensitivity in detecting radiographic OA progression. Osteophyte formation is often one of the first indicators of OA progression, a parameter scored with the OARSI and Kallman methods. Consistent with the limited available literature, all of the methods we used showed good interobserver and intraobserver reliability.^33,34^ While there is a recommendation for longitudinal studies to involve 2 experienced observers and assess radiographs in pairs to improve reproducibility and sensitivity, we opted to assess the radiographs randomly and in a blinded fashion by 3 hand surgeons.^32^

A key strength of this study is its long-term follow-up and the longitudinal comparison of mid-term postoperative results within the same cohort. The development of clinically measurable PTOA occurs over a highly variable time course, ranging from a few years to several decades, depending on the severity of the articular injury.^4^ In our study, the mean follow-up duration was 16 years, which can be considered a sufficient time frame for detecting OA progression. Notably, there is evidence that progressive hand OA can be detected as early as 2 years post-injury.^35^

Our study has several limitations. First, it is a retrospective design with a relatively small sample size, which may limit the statistical power. Second, we only included cases of unstable dorsal fracture-dislocations treated with extension block pinning that were shown to be in subluxation and suitable for extension block pinning regarding fracture pattern and time of injury (ie, acute). The original cohort consisted of 53 patients treated between 2000 and 2009, of whom 25 attended follow-up evaluations in both 2010 and 2021, introducing potential selection bias as the dropouts could interfere the results either way. Third, the radiographic assessments were based on plain radiographs, whereas modern imaging techniques such as computed tomography and magnetic resonance imaging now play a crucial role in OA research, offering more detailed insights into joint pathology.^36-38^

In conclusion, the long-term functional outcome following surgical treatment of dorsal PIP joint fracture-dislocations remained stable throughout the follow-up period, despite the observed progression of radiographic OA. Key prognostic factors for decreased AROM and radiographic OA progression included residual articular step-off and patient age more than 45 years. While dorsal PIP joint fracture-dislocations are commonly considered to be associated with prolonged pain, stiffness, long-term functional impairment, and osteoarthritis in the literature, these findings challenge some of the established beliefs surrounding the condition. They emphasize the need to carefully consider factors such as articular step-off and patient age as patients above 45 years might benefit less from extension block pinning in terms of long-term outcomes.

## Supplemental Material

sj-docx-1-han-10.1177_15589447261424449 – Supplemental material for A 16-Year Longitudinal Study of Long-term Clinical and Radiographic Outcomes in Dorsal Proximal Interphalangeal Joint Fracture-Dislocations Supplemental material, sj-docx-1-han-10.1177_15589447261424449 for A 16-Year Longitudinal Study of Long-term Clinical and Radiographic Outcomes in Dorsal Proximal Interphalangeal Joint Fracture-Dislocations by Panu H. Nordback, Marjut Westman and Eero Waris in HAND
