# Supplementary material for: A 16-Year Longitudinal Study of Long-term Clinical and Radiographic Outcomes in Dorsal Proximal Interphalangeal Joint Fracture-Dislocations
Source: Hand (N Y). 2026 Mar 16:15589447261424449. Online ahead of print. doi: 10.1177/15589447261424449 (PMC12995736; doi:10.1177/15589447261424449)

**Supplementary figure 1.** Lateral radiographs of a 62-year-old woman who sustained a dorsal fracture-dislocation of the fifth finger: (a) initial injury, (b) post-treatment with an extension block pin and percutaneous intramedullary tamping of the impacted articular fragment, (c) 4 years postoperatively, and (d) 15 years postoperatively. At the final follow-up, there was an active extension deficit of 15° and active flexion of 70°, but the patient was satisfied with the outcome.


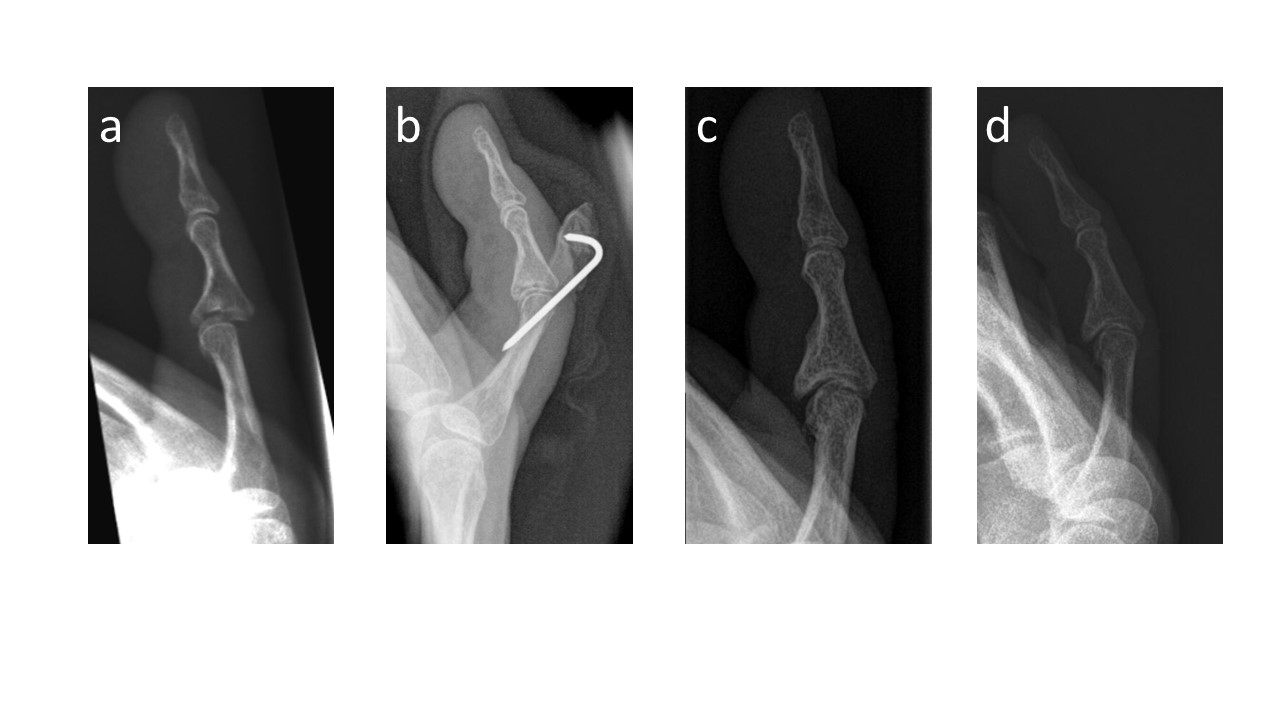


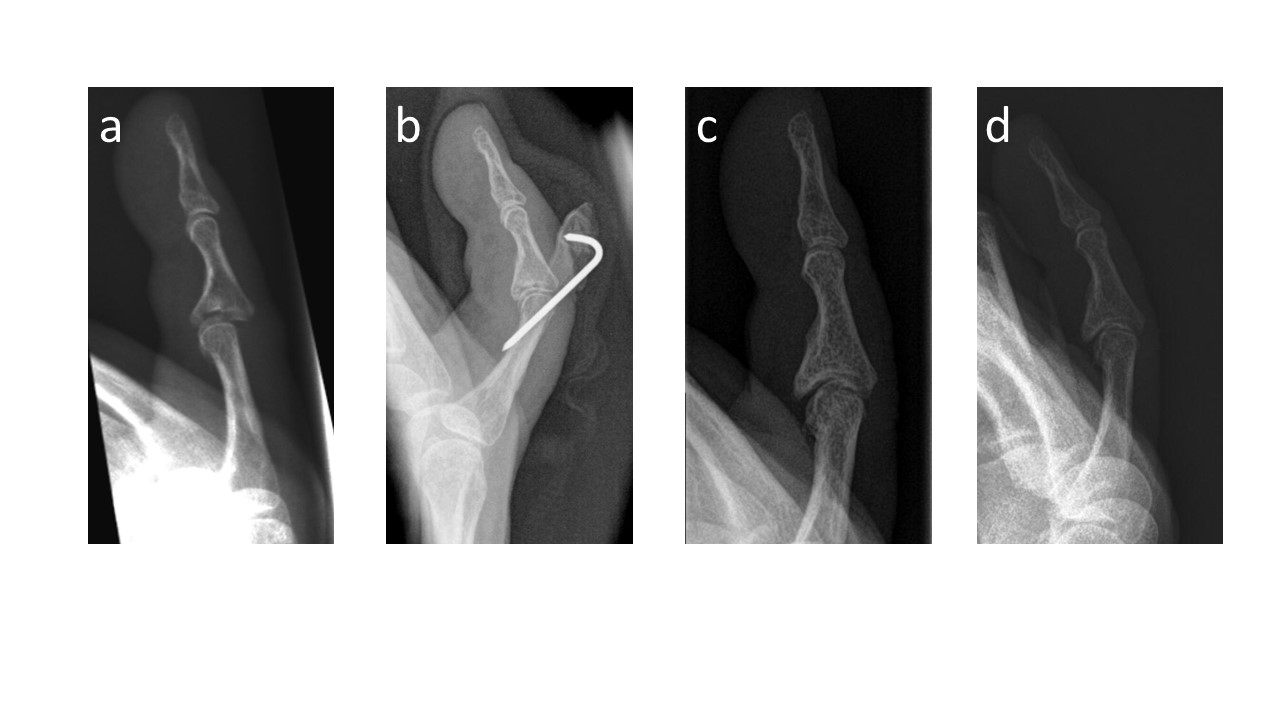

Supplement: sj-docx-1-han-10.1177_15589447261424449 – Supplemental material for A 16-Year Longitudinal Study of Long-term Clinical and Radiographic Outcomes in Dorsal Proximal Interphalangeal Joint Fracture-Dislocations [file sj-docx-1-han-10.1177_15589447261424449.docx]
